# A Review: *Haemonchus contortus* Infection in Pasture-Based Sheep Production Systems, with a Focus on the Pathogenesis of Anaemia and Changes in Haematological Parameters

**DOI:** 10.3390/ani12101238

**Published:** 2022-05-11

**Authors:** Kate J. Flay, Fraser I. Hill, Daniela Hernandez Muguiro

**Affiliations:** 1 Department of Veterinary Clinical Sciences, Jockey Club College of Veterinary Medicine and Life Sciences, City University of Hong Kong, Kowloon Tong, Hong Kong, China; 2 CityU Veterinary Diagnostic Laboratory Co., Ltd., City University of Hong Kong, Kowloon Tong, Hong Kong, China; fraser.hill@cityu.edu.hk (F.I.H.); daniela.hernandez@cityu.edu.hk (D.H.M.)

**Keywords:** parasite, barbers pole, small ruminant, nematode, gastrointestinal parasite, worm

## Abstract

**Simple Summary:**

Infection with *Haemonchus contortus* parasites (haemonchosis) is an important cause of anaemia in sheep. Haemonchosis is a global problem, although sheep that are kept in warm, high rainfall environments are at the greatest risk of infection due to the favourable conditions for *H. contortus* survival. Following ingestion, the parasites develop in the abomasum of sheep. Various factors such as age, breed, health, nutritional status, and larval challenge influence the severity of clinical disease. Hyperacute, acute, and chronic haemonchosis are reviewed, focusing on the pathophysiology of haemonchosis, associated clinical signs, and haematological and biochemical findings.

**Abstract:**

Haemonchosis is an important cause of anaemia in sheep worldwide, particularly those that are kept in pasture-based systems in warm, high rainfall environments. Potential outcomes vary based on the severity of infection and the sheep’s immune response, however, in some sheep infection can lead to death. The consequences of *Haemonchus contortus* infection mean that it has been well-studied in a range of different farming systems. However, to our knowledge, there has not been a recent review focused on the pathophysiology of anaemia caused by haemonchosis. Thus, this review provides an in-depth discussion of the literature related to the pathophysiology of haemonchosis and associated clinical signs for hyperacute, acute, and chronic haemonchosis. Additionally, haematological and biochemical findings are presented, and various diagnostic methods are assessed.

## 1. Introduction and Epidemiology

Many diseases can contribute to the development of anaemia in sheep. Haemonchosis, defined as infection with the nematode parasite *Haemonchus contortus*, is one such disease. It is recognised as one of the most significant causes of anaemia in small ruminants of all ages in many countries throughout the world [1,2].

*H. contortus* is a nematode parasite of the Phylum: Nematoda, Class: Secernentea, Order: Strongylida, and Family: Trichonstrongylidae. *H. contortus* is adapted to a wide range of climatic zones but survives best in warm, high rainfall environments. Traditionally, haemonchosis was more commonly seen in known high-risk areas, however climate change and warming of new areas of the planet appear to be allowing *H. contortus* to survive and flourish in previously low-risk zones [2]. Therefore, haemonchosis should be included on the differential diagnosis list when investigating cases of anaemia or death in sheep, irrespective of geographical location.

A warm, moist enviro nment is needed for the free-living stages of *H. contortus* to survive and develop outside the host [3,4]. If suitable conditions are present in any season, haemonchosis can occur [5]. In the tropical climate zones between 23.5° North and South, such as South-East Asia, Southern India, Central Africa, and America and Northern South America, *H. contortus* survives well and disease prevalence is highest [6]. Sub-tropical regions immediately north and south of 23.5° N and 23.5° S, including parts of Australia, Southern Africa, Southern North America, South America, and South-eastern China have warm, humid, summer-dominant rainfall, providing suitable environments for *H. contortus* survival [6]. Warm temperate zones beyond 40° latitude in New Zealand, Northern Europe, Scandinavia, North Asia, and North America have suitable conditions in wet summers and autumns for larval survival, while temperate zones between 45° and 65° latitude in Sweden, France, Denmark, and the Netherlands are too cool most of the year to permit larval survival and development [6]. Haemonchosis is rare in arid regions of the world as there is insufficient moisture for the free-living larval stages of *H. contortus.* However, increased rainfall or irrigation can allow larvae to survive in warmer arid areas [1,7]. Additionally, important is the ability of the fourth larval stage of *H. contortus* to undergo hypobiosis (arrested development), contributing to their ability to survive in cold or arid environmental conditions [8,9].

Optimal conditions for *H. contortus* egg hatching and larval development occur at ambient vegetation microclimate temperatures of 22 to 26 °C and humidity close to 100% [10,11,12]. During dry weather, larvae may remain in desiccated faeces and emerge after rain, contributing to a surge in infection [13]. If ideal environmental conditions are present, development from the egg to infective third stage larvae (L3) can occur within four days [6], otherwise, larval development time is more variable. Low humidity and desiccation rapidly kill both eggs and larvae [14].

The ensheathed L3 larvae retain the cuticle of the second-stage larvae and are the most resistant, free-living form, able to survive for long periods if the temperature and humidity are favourable. The L3 move out of the faeces, onto the soil, then randomly migrate horizontally and vertically up the grass sward independent of free water [15]. The larvae do not feed, relying on stored energy for survival [13]. In cold conditions (above freezing) larvae are inactive, consume little energy, and can survive for long periods of time, allowing larvae to survive the winter in some climates. In tropical climates the survival of L3 is typically less than five weeks, as the larvae are more active and consume energy stores [6]. This provides an opportunity for spelling of pastures (i.e., periods without grazing) to be used as a control method in some geographical regions [16].

## 2. Pathogenesis

The L3 larvae are the infective stage of *H. contortus* and are consumed when the animals graze the pasture sward. Once ingested, a range of factors derived from the host sheep will influence survival and establishment of the L3 larvae within the abomasum. These include age, breed, health, and nutritional status of the sheep, as they can impact an individual’s ability to mount immune reactions to the *H. contortus* larvae [17].

### 2.1. Parasitic Stages of H. contortus

After ingestion by susceptible sheep, the L3 differentiate, and by the third day after infection, complete the first parasitic ecdysis (cuticle shedding), becoming the fourth larval stage (L4) [3]. By days seven to nine, the L4 can be up to 5 mm in length and a second ecdysis has taken place, forming the fifth larval stage (L5) [3]. Around days ten to eleven, just before the final ecdysis, the immature L5 develop a lancet in their buccal cavity [18] and burrow through the mucous layer on the surface of the abomasum [19] into the gastric pits of the abomasal mucosa [20]. Once in the gastric pits, the L5 use the lancet to cut the abomasal tissue and induce haemorrhages [21] before ingesting the leaking blood [22]. The L5 larvae then mature into adult forms, continue feeding, and begin sexual differentiation and reproduction. Adult *H. contortus* females begin to produce eggs from day 12 to 15 and have a high biotic potential [3].

### 2.2. Syndromes of H. contortus

The pathophysiology of haemonchosis and associated clinical signs are primarily associated with anaemia and hypoalbuminemia. The magnitude and consequences of the anaemia and hypoalbuminemia depend on the parasite burden, their stage of development, the age and size of the infected sheep, and the host’s response. Haemonchosis can been categorized into a continuum of three stages, including hyperacute, acute, and chronic [1].

#### 2.2.1. Hyperacute Haemonchosis

Hyperacute haemonchosis is rare and occurs in animals exposed to heavy burdens of *H. contortus* (up to 30,000 larvae per animal) over a short period of time. It is more common in young animals. Death is sudden with either no previous clinical signs, or anaemia and melaena may be detected in surviving animals [1,23].

In lambs, haemoglobin concentrations below 30 g/L have been associated with mortality [24]. Infected sheep can lose 0.2–0.6 L of blood per day, an approximate equivalent of 18–54 g of haemoglobin per day [25] and can exsanguinate within a week before compensatory erythropoiesis occurs [1,26]. The volume of blood lost in this time may represent half of the blood volume of a lamb. Although the pathogenesis of death for hyperacute haemonchosis has not been clearly described, it is reasonable to assume that death can be attributed to hypovolemic shock. Severe haemorrhage causes a rapid decrease in the circulating blood volume and blood pressure, culminating in hypovolemic shock where tissue perfusion is insufficient to sustain aerobic metabolism [27]. Hypoxia impairs mitochondrial respiration and the production of adenosine triphosphate (ATP). Without ATP to power the ion pumping system, membrane depolarisation occurs, calcium floods into the cell and activates calcium-dependent phospholipases and proteases, thus resulting in uncontrolled cell swelling, hydrolysis of cellular components, cell death, and necrosis [28]. Supporting tissue hypoperfusion and therefore hypovolemic shock as the cause of death, a study identified necrosis around the central veins of the liver of sheep dying from anaemia [29]. Hepatocytes around the central veins are furthest from the oxygenated blood of the hepatic artery and therefore more susceptible to hypoxic injury [30].

#### 2.2.2. Acute Haemonchosis

Acute haemonchosis is characterized by lower burdens of *H. contortus* (approximately 2000–20,000 larvae per animal), resulting in a less severe loss of blood and anaemia that develops over a longer period of time, compared to hyperacute haemonchosis. In acute haemonchosis, sheep show clinical signs associated with anaemia and hypoproteinemia such as lethargy, weakness, increased respiratory and heart rates, and pale mucous membranes. There is an initial decrease in packed cell volume (PCV), but compensatory erythropoiesis occurs within the first 14 days of infection, and full compensation and apparent recovery is seen over a period of six weeks after infection [31].

In most mammals, including ruminants, compensatory erythropoiesis can be detected three to four days after the onset of haemorrhage or haemolysis [32]. Compensatory erythropoiesis continues until the animal’s baseline PCV is reached. A threefold increase in the rate of erythropoiesis and iron utilization has been described in sheep infected with *H. contortus* [31]. It can be assumed, therefore, that this compensatory process may result in an initial steady normalization of the infected sheep’s PCV as was described [31]. However, the continuous loss of blood over a period of weeks outpaces iron reabsorption in the intestine, with the progressive depletion of iron stores leading to a state of iron deficiency [31].

Iron is vital for the formation of the haem fraction of haemoglobin in red cells. Total body iron is distributed among several compartments. Approximately 60–70% of iron is present in haemoglobin within red blood cells (RBC), 20–30% is stored as ferritin or haemosiderin in macrophages and hepatocytes, 3–7% in myoglobin, 1% in enzymes, and 0.1% is undergoing transport bound to transferrin in plasma [33]. Iron for steady state erythropoiesis comes from three sources: dietary iron, recycling from senescent RBC, and storage from hepatocytes [34].

Dietary ferrous iron (Fe^2+^) is mainly absorbed in the duodenum where it is transferred across the enterocytes by the enzyme ferroportin, before binding to transferrin in the plasma for transport via the circulation for use or storage [35]. Iron homeostasis is tightly controlled to avoid excess iron absorption (and subsequent toxicity) while still providing sufficient iron for haemoglobin production. When liver iron stores are high, the enzyme hepcidin is released by hepatocytes. Hepcidin binds to, and destroys, intestinal ferroportin; hence, any absorbed iron remains within the enterocytes and is lost as the enterocytes are shed in the intestinal lumen [35]. When iron stores are low, hepcidin production is decreased, thereby increasing ferroportin action and iron absorption [35].

In sheep, RBC have an average lifespan of 120 days [36]. Eventually, aged RBC are recognised as degenerate and are phagocytosed by macrophages. Haemolysis of senescent RBC mainly occurs by macrophages in the spleen, and to a lesser degree, in liver and bone marrow [32]. During haemolysis, haemoglobin is degraded into bilirubin, amino acids, and iron. Bilirubin is degraded or excreted, and the amino acids and iron are recycled. Iron may be stored within macrophages or transported to other tissues for use, including the bone marrow for erythropoiesis. Iron stored as ferritin in macrophages, hepatocytes, intestinal mucosal epithelial cells, and RBC precursors is relatively soluble, and constitutes a mobile source of iron. Hemosiderin found in lysosomes of macrophages in spleen, liver, and bone marrow is relatively insoluble and constitutes a poorly mobile source of iron. Iron from storage, haemolysis, and the diet is transported by transferrin to RBC precursors, which incorporate iron into haem and then haemoglobin [32]. During haemolysis and internal haemorrhage, virtually all the amino acids and iron are recycled. However, in external haemorrhage, these components are lost and require replenishment.

Iron deficiency can result from insufficient intake, inadequate absorption of iron from the intestinal tract, or chronic external blood loss. Iron deficiency is defined as the reduction in total body iron and may progress over three sequential stages [35]. The first stage is storage iron depletion. Whenever iron is lost, the bone marrow stores are the first to be affected in order to meet organic requirements. At this stage, erythropoiesis is unaffected. In the second stage, there is iron-deficient erythropoiesis. When bone marrow iron stores are depleted and transport iron is low, iron-deficient RBC are produced, and the PCV drops but remains within normal limits. In the third stage, there is iron deficiency anaemia. The PCV drops below normal limits, and initially, the anaemia is regenerative. However, as the iron deficiency worsens, a microcytic hypochromic anaemia manifests, and the regeneration becomes inadequate for the degree of anaemia [35].

It is to be expected that in order to maintain iron stores and meet the concurrent threefold increase in erythropoiesis and iron demand, sheep infected with *H. contortus* need to increase iron input from the diet or supplements. It has been shown that sheep with haemonchosis have limited ability to reabsorb iron from RBC lost into the gastrointestinal tract following the feeding activity of *H. contortus* [31]. However, intramuscular iron supplementation of lambs experimentally infected with *H. contortus* reduced the severity of the anaemia and increased iron stores in the bone marrow [37]. Lambs infected with *H. contortus* and supplemented with iron had higher RBC counts, hematocrits, and haemoglobin concentrations than infected lambs without iron supplementation [37].

Haemonchosis causes concurrent hypoproteinemia [38]. This hypoproteinemia occurs due to a variety of mechanisms, including blood loss, haematophagous activity of *H. contortus*, leakage of protein into the abomasal lumen through disrupted cell junctions and increased permeability [39], decreased protein absorption due to abomasal epithelial cell loss, tissue reparation, and increased mucus production [39]. There is also increased protein use to repair damaged tissues and mount an immune response [40].

**Figure 1:**
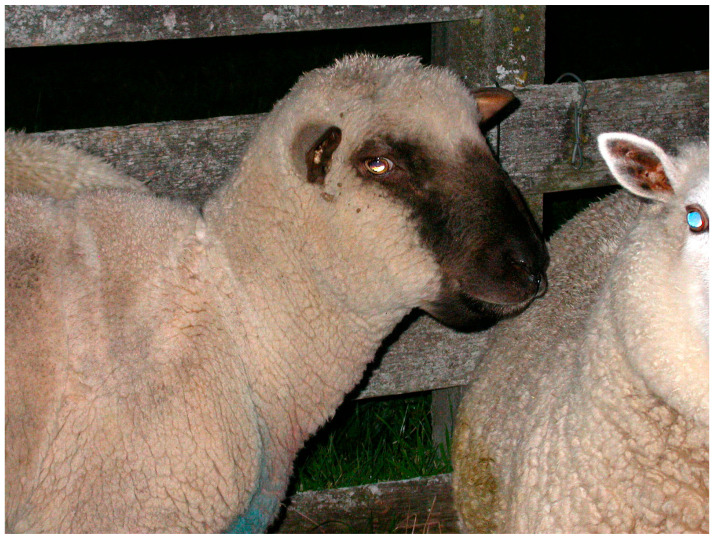
Oedema in the intermandibular space of a lamb (known as “bottle jaw”). *Image courtesy of Professor K G Thompson, Massey University, New Zealand*.

Hypoalbuminemia results in decreased intravascular oncotic osmotic pressure and oedema that may be generalized or localized in the intermandibular space (referred to as “bottle jaw”, Figure 1) or cervical region [18,23]. Plasma proteins, mainly albumin, create an oncotic draw that helps maintain fluid within the blood vessels. Fluid will shift out of the vascular space if the amount of albumin in plasma is significantly decreased (usually <15 g/L) [32]. Oedema can form in any organ or tissue, but it is most commonly seen in subcutaneous tissue and more obvious in regions with high hydrostatic pressures, or in lower parts of the body related to the influence of gravity. These factors may explain the formation of oedema in the intermandibular space seen in acute haemonchosis, although some sheep may die before the development of oedema [23].

#### 2.2.3. Chronic Haemonchosis

In chronic or subclinical haemonchosis, sheep ingest lower numbers of H. contortus and infections may go unnoticed. Chronic haemonchosis typically occurs in environments not suitable for the development of the infective larvae during less favourable periods in seasonally endemic zones, or where there are effective control measures to prevent acute haemonchosis [23].

Infection may manifest as a reduction in productivity, feed conversion, growth rate, or milk yield [41]. Disease may become clinically overt when worm burdens increase or with poor nutritional conditions.

### 2.3. Abomasal Pathology

The pathology in the abomasum is varied. The initial changes are a loss of surface mucous, despite hyperplasia of the mucous producing cells, along with multiple haemorrages and congestion of mucosal blood vessels [42]. Mast cells and eosinophils infiltrate the lamina propria of the abomasum in response to the presence of the parasite [43,44,45], while immunoglobulin A is increased in the mucous [45]. Later, mucous content will return to normal, but there is oedema of the mucosa and submucosa [42].

Digestion is interfered with due to changes in the composition of the cell types making up the abomasal mucosa, resulting in altered digestive efficiency. Abomasal mucosal effects include loss of acid-secreting parietal cells, hyperplasia of gastric pits with less mucin production, and increased mucous neck cells [46]. Parietal cells control the fate of other cells in the mucosa and synthesise growth factors, so their loss leads to a decrease in gastric acid secretion, increased pH, hypergastrinaemia, and hyperpepsinogenaemia [47].

### 2.4. Larval Challenge

The number of *H. contortus* nematodes in the abomasum, their stage of development, and the age and size of the infected sheep all determine the severity of anaemia. For young lambs <20 kg live weight, 112 intra-abomasal *H. contortus* nematodes can result in haemoglobin concentrations of <105 g/L, while 355 nematodes can reduce haemoglobin to <80 g/L [48]. However, in sheep >50 kg live weight, 1259 nematodes were needed to decrease haemoglobin to <80 g/L [48]. Additionally, there was wide variation in these parameters between individual sheep, with some having haemoglobin concentrations as low as 36 g/L with a total worm count of only 100 [48].

If sheep are infected with large numbers of *H. contortus* nematodes, then blood loss can exceed the ability of erythropoiesis to respond and terminal anaemia can result [1]. In one study, sheep were experimentally infected with 7400 infective *H. contortus* larave and lost 0.25 L of blood per day into the abomasum, contributing to a marked fall in PCV to only 11% by 40 days post-infection [39]. In addition, there was a decrease in digestive efficiency, and despite recycling of nitrogen from the digested blood, there was also a net loss of amino acids [39]. In another study of 3 to 5-month-old lambs infected with 11,000 *H. contortus* larvae, the PCV decreased over five weeks to an average of 23.3% (range 19.6–30.9%), and serum iron concentrations decreased to an average of 0.95 mg/L (range 0.5–1.5 mg/L) [49], whereas the normal sheep serum iron reference interval is 1.66–2.22 mg/L [50].

A further study, where 7-month-old lambs were given three infections of 10,000 *H. contortus* larvae at 8-week intervals, found the mean RBC concentrations fell from pre-infection concentrations of 11 *×* 10^9^/L to 6 × 10^9^/L after seven weeks of infection [51]. Study lambs were also sacrificed during the experiment. Of the larvae used as the infecting dose, only between 0 to 36% could be recovered as adults 24 weeks later, despite no anthelmintic treatments being given [51]. Additionally, after the initial fall in RBC concentrations (to week seven), these then increased back to near normal values despite repeated infections, indicating the capacity of the lambs to develop resistance to infection [51]. This is consistent with the self-cure phenomenon described previously [31].

## 3. Diagnosis

Antemortem methods for confirmation of *H. contortus* infections include testing for anaemia and confirming the presence of parasites. While detection of *H. contortus* by faecal egg counts and larval culture are a conventional method for diagnosis [23], by the time egg-laying adults develop, affected sheep can already be anaemic.

The total blood volume of a lamb at birth has been determined to be 0.17 L per kilogram of bodyweight, dropping to about 0.1 L/kg by day 30 of age [52]. A decrease in the concentration of RBC, haemoglobin, and PCV can all be defined as anaemia. The normal haematological reference range values for sheep are listed in Table 1.

### 3.1. Haematology and Selected Biochemical Findings

A range of changes may be seen on a complete blood count (CBC). Anaemia may be mild to severe and regenerative to non-regenerative. With compensatory erythropoiesis (as seen in acute haemonchosis), the anaemia may be macrocytic and hypochromic. With chronic blood loss, the anaemia may become microcytic, hypochromic, and non-regenerative due to iron deficiency. The leukocyte changes are variable; an inverse relationship between worm burden and total leukocyte count has been demonstrated [53]. Lymphopenia is common, but the total leukocyte count may be within normal limits or increase due to an increase of neutrophils and eosinophils [53]. Leukopenia characterized by lymphopenia and neutropenia may occur and may be secondary to migration of the leukocytes into the abomasum where the *H. contortus* and lesions are located, or due to immune suppression [54].

Serum iron and protein concentrations both decrease [31]. Early investigations of the loss of RBC using radioisotopes of iron (Fe^59^) showed parasitised sheep lost more iron than non-parasitised sheep [55,56]. The hypoproteinemia may be characterized by low albumin and globulin. This can be attributed to blood loss, protein loss, decreased protein intake, and protein catabolism. Serum protein losses of 10–12 g/L have been recorded in experimental *H. contortus* infections, from pre-infection concentrations of 62 g/L down to 50 g/L, 20 weeks after infection [57]. Albumin is the principal protein lost, while globulin concentrations may remain in the normal range [54]. This may be due to a combination of the selective loss of the smaller molecule albumin and an increase in globulin, as immunoglobulins are produced in response to inflammation caused by the parasite infection and tissue damage [58].

Serum immunoglobulins IgG and IgM may be decreased in sheep with heavy burdens of *H. contortus* worms. Experimentally infected sheep with heavy worm burdens show significantly lower IgG (9.36 ± 0.16 g/L) and IgM (0.59 ± 0.01 g/L) concentration compared to controls (IgG 10.35 ± 0.09 g/L, IgM 0.83 ± 0.01 g/L) and sheep with light (IgG 10.22 ± 0.05 g/L; IgM 0.74 ± 0.01 g/L) and moderate (IgG 10.12 ± 0.03 g/L; IgM 0.67 ± 0.01 g/L) burdens of *H. contortus* [54]. This was attributed to translocation of immunoglobulins into the lumen of abomasum to protect against *H. contortus* [54].

### 3.2. FAMACHA Score

Many options are available for assessing sheep for anaemia [59]. Some of these, such as CBC, PCV measurement, and evaluation of MCV, MCHC, and reticulocyte counts [59], require blood samples to be collected from the sheep and access to suitable laboratory diagnostic equipment. This may not be practical in many on-farm situations, particularly where rapid diagnosis is required. Therefore, a practical method allowing for anaemia classification in the field was developed in South Africa, based on conjunctival mucous membrane colour in small ruminants [60]. The FAMACHA system (named after the developer Dr Faffa Malan (FAffa MAlan CHArt)) was developed by comparing the colour of the conjunctiva (on a scale of 1–5 with various stages of anaemia related to haemonchosis), with their measured PCV [60]. A value of 1 (red) is considered non-anaemic and a PCV of 35%, 2 a PCV of 25%, 3 a PCV of 20%, 4 a PCV of 15%, through to a value of 5 (white) and a PCV of 5%, being severely anaemic [61].

Other single diagnostic methods for diagnosis of haemonchosis in live sheep include measurement of the presence of ecto-adenosine deaminase (E-EDA) activity in RBC [62,63] and faecal occult blood tests [62,64]. However, none of these methods appear to offer distinct advantages over basic haematology or the FAMACHA chart.

### 3.3. Post-Mortem Diagnosis

**Figure 2:**
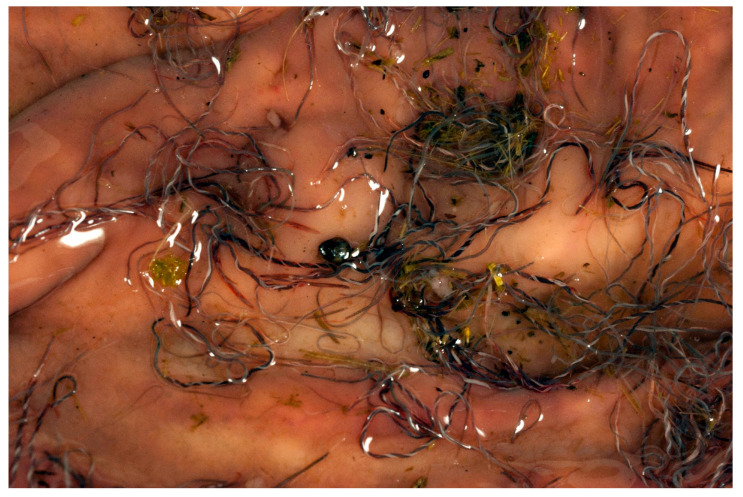
*Haemonchus contortus* parasites in the abomasum of an affected lamb, demonstrating the characteristic red and white twisted “barber’s pole” appearance. *Image courtesy of Professor K G Thompson, Massey University, New Zealand*.

Post-mortem examination of dead sheep is another rapid, accurate method of confirming a diagnosis, as it allows for visualization of the *H. contortus* parasites in the abomasum of affected animals. The parasites have a characteristic red and white, twisted “barber’s pole” appearance related to the blood-filled intestine spiralling around the white reproductive tract of the female nematodes, as seen in Figure 2.

### 3.4. Molecular Tools for Diagnosis

Molecular methods for diagnosis are providing new options for testing for the presence of *H. contortus* but have limitations due to the inhibition effects of faeces on the polymerase chain reaction (PCR) and their accessibility to veterinarians in the field [2]. Development of suitable PCR tests is on-going, with droplet digital PCR showing promise [65]. In a comparison of diagnostic methods, molecular tests had the greatest sensitivity and were able to identify *H. contortus* to the species level, while faecal egg counts using the McMaster method had a high specificity but a lower sensitivity than molecular methods [66].

## 4. Conclusions

Anaemia in sheep related to infection with *H. contortus* remains a significant challenge in sheep production systems. Understanding the complexities of blood loss and erythropoiesis is essential to anticipating the likely effects of significant infections. Minimizing ingestion of infective larvae, anticipating their maturation and effects on sheep, treating in the timeliest manner, and having effective diagnostic tools in place will assist in mitigating potential losses from infection.

## Figures and Tables

**Table 1:** Normal haematological reference range values for sheep, adapted from [50]. JOHN WILEY AND SONS LICENSE TERMS AND CONDITIONS.

Value	Abbreviation	Reference Range	Unit
Haemoglobin	Hb	90–150	g/L
Haematocrit (packed cell volume)	PCV	0.27–0.45	L/L
Red blood cells	RBC	8.0–18.0	×10^12^/L
Mean cellular volume	MCV	28.0–40.0	fL
Mean cellular haemoglobin	MCH	8.0–12.0	pg
Mean cellular haemoglobin concentration	MCHC	310–340	g/L

## Data Availability

No new data were created or analyzed in this study. Data sharing is not applicable to this article.
